# Multimodal treatment of glioblastoma with multiple lesions - a multi-center retrospective analysis

**DOI:** 10.1007/s11060-024-04810-3

**Published:** 2024-11-19

**Authors:** Harald Krenzlin, Dragan Jankovic, Alice Dauth, Felipa Lange, Martin Wetzel, Leon Schmidt, Insa Janssen, Christoph Richter, Marcus Stockinger, Heinz Schmidberger, Marc A. Brockmann, Clemens Sommer, Bernhard Meyer, Naureen Keric, Florian Ringel

**Affiliations:** 1 Department of Neurosurgery, University Medical Center, Gutenberg University Mainz, Mainz, Germany; 2 Department of Neuroradiology, University Medical Center, Gutenberg University Mainz, Mainz, Germany; 3 Department of Radiation Oncology and Radiation Therapy, University Medical Center, Gutenberg University Mainz, Mainz, Germany; 4 Institute of Neuropathology, University Medical Center, Gutenberg University Mainz, Mainz, Germany; 5 Department of Neurosurgery, University Medical Center, Technical University of Munich, Munich, Germany; 6 Department of Neurosurgery, University Medical Center Mainz, Johannes Gutenberg University of Mainz, Langenbeckstr. 1, 55131 Mainz, Germany

**Keywords:** Multifocal, Multicentric, Glioblastoma, Multimodal therapy, Extent of resection

## Abstract

**Objective:**

The presence of multiple localizations (ML) in glioblastoma is rare and associated with perceived poor prognosis. The aim of this study is to evaluate the impact of a multimodal treatment on progression-free survival (PFS) and overall survival (OS) in ML glioblastoma.

**Methods:**

Patients presenting with CNS WHO grade 4 glioblastoma with ML to 2 major German Departments of Neurosurgery between January 1st, 2008, to December 31st, 2020 were included in this study. Primary outcome parameters were extent of resection (EOR) using the 2021 RANO criteria, progression free- and overall survival.

**Results:**

A total of 483 patients with newly diagnosed glioblastoma (CNS WHO grade 4) were assessed. 134 patients presented with ML (72 multifocal (MF), 62 multicentric (MC)). The median PFS and OS did not differ among MC and MF glioblastomas. The EOR was a significant predictor of PFS and OS in ML glioblastoma. complete-, near total-, and subtotal resection significantly prolonged PFS (*p* < 0.0001) and OS (*p* < 0.0001) compared to biopsy alone. Standard radiotherapy (*p* = 0.045) and hypofractionated (*p* < 0.0001) radiotherapy and adjuvant treatment (Stupp protocol) prolonged PFS (*p* = 0.0012) and OS (*p* < 0.0001). In multivariate analysis Karnfosky performance score, EOR, and concomitant adjuvant treatment remained significant factors influencing OS. Propensity score matching of patients with ML and solitary lesion tumors showed similar PFS and OS (*p* = 0.08).

**Conclusion:**

The presented data suggests that glioblastomas with multiple lesions treated with multimodal therapy equal survival rates compared to patients with solitary lesion tumors can be achieved. The results reflect the importance of an equally aggressive maximal treatment effort in this particular and often marginalized group of patients.

**Supplementary Information:**

The online version contains supplementary material available at 10.1007/s11060-024-04810-3.

## Highlights

• Multifocal and multicentric glioblastoma are the same entity according to their clinical course.

• EOR and adjuvant treatment are the most important predictors of PFS and OS in multiple lesion glioblastoma.

• Multiplelesion- and solitary lesion glioblastoma have similar survival rates if treated equally.

## Introduction

Glioblastoma is a devastating and mostly fatal disease. The median progression-free (PFS) and overall survival (OS) is dismal at only 7.4 months and 15 months respectivly [1–3]. These tumors either occur as solitary lesion (SL) or with multiple lesions (ML) at the time of diagnosis [4, 5]. The reported incidence of glioblastoma with multiple lesions (ML) ranges between 2–35%.^5^ OS is believed to be worse for glioblastoma with ML [6]. In previous analyses only about 17% survive one year or longer with a median OS of 8 months [6, 7]. ML tumors can be further distinguished based on the presence or absence of an imaging connection between contrast enhancing lesions [8]. Those with a visible imaging connection, e.g. connection in fluid-attenuated inversion recovery (FLAIR) magnetic resonance imaging (MRI), are termed multifocal (MF). Those tumors with lesions within separated areas that show no imaging connection are termed multicentric (MC) [9, 10]. Tumor heterogeneity is considered a hallmark of glioblastoma, and tumor cell plasticity contributes to the complexity [11]. MF tumors genetically resemble solitary glioblastoma [4, 12]. Analysis of multiple foci from a single patient revealed monoclonal origin [12]. However, MF tumors have a higher frequencies of epidermal growth factor receptor (EGFR) mutations and the co-occurrence of EGFR/ phosphatase and tensin homologue (PTEN) alterations [4, 12]. In contrast, lesions in MC glioblastoma are more often genetically distinct and present a rather metachronous, independent glioma development [13]. The high incidence of genetic alterations in key pathways such as EGFR, PTEN, telomerase reverse transcriptase (TERT) and p53 are thought to be responsible for a highly malignant and invasive phenotype in all of these tumors [4, 12, 13].

In glioblastoma, standard treatment consists of maximal safe surgical resection, radiotherapy (RT), and alkylating chemotherapy with temozolomide (TMZ) [14]. Greater extent of resection (EOR) is associated with improved survival in high-grade gliomas [2–7] [15–17]. Gross total resection (GTR) of more than 98% of tumor volume is superior to lesser degrees of resection [17, 18]. In addition, EOR improves the efficacy of adjuvant radiation and chemotherapy by reducing disease burden and improving chemotherapy patency and longevity [18]. Treatment of tumors with multiple lesions is less clear. Due to safety reasons, RT is often omitted for larger target volumes [7, 19]. Although the safety of fractioned RT and concomitant chemotherapy in patients with ML has recently been demonstrated, the benefit for survival remains uncertain [7]. Furthermore, the majority of surgeons would be reluctant to offer surgical resection in ML glioblastomas. However, in a cohort of 34 patients resection of the largest contrast-enhancing lesion was shown to be beneficial for OS compared to biopsy alone [16]. A larger series of 82 MF and 18 MC glioblastoma also hinted superiority of larger EOR in both entities [9]. However, stringent and conclusive data on the EOR and the influence of multimodal therapy in ML glioblastoma are still missing [6].

Therefore, the aim of this study was to evaluate the role of multimodal treatment in MF and MC glioblastoma and its correlation with PFS and OS. In addition, the clinical course is evaluated in comparison to unifocal glioblastoma.

## Materials and methods

### Study design and setting

Patients presenting with CNS WHO grade 4 glioblastoma with ML to the Department of Neurosurgery, Technical University of Munich and to the Department of Neurosurgery, University Medical Center, Gutenberg University Mainz, between January 1st, 2008, to December 31th, 2020 were retrospectively analyzed.

The article was drafted based on The Strengthening the Reporting of Observational Studies in Epidemiology (STROBE) recommendations (https://www.equator-network.org/reporting-guidelines/strobe/).

## Patients and parameters

Inclusion: Patients over 18 years of age with newly diagnosed glioblastoma CNS WHO grade 4 were included in our study.

Exclusion: Patients with previous treatment for glioma were excluded.

Baseline characteristics: Age, sex, functional neurological status at admission and discharge using the Eastern Cooperative Oncology Group (ECOG) performance status, as well as radiological and molecular tumor features, were analyzed. All patients underwent either biopsy or tumor resection.

Outcome parameters: The 2021 RANO categories for EOR in glioblastoma were applied to early (< 72 h) postoperative magnetic resonance imaging (MRI) to determine the extent of tumor removal [20]. Using the adapted RANO resection criteria, complete resection (CR) was defined as resection of all contrast-enhancing (CE) tumor, near total resection (NTR) as 95–99.9% CE tumor reduction ± ≤1 cm [3]. residual CE tumor, subtotal resection (STR) as 80–94.9% CE tumor reduction + ≤5 cm [3]. residual CE tumor, partial resection (PR) as < 80% CE tumor reduction ± >5 cm [3]. residual CE tumor (for mass effect-related symptoms); and biopsy as no tumor reduction (procedure performed for tissue-based diagnosis only) [20]. PFS and OS were defined from the time of surgery to radiographic progression and death, respectively [21]. Response criteria established by the RANO working group were used to define progression [22].

### Statistics

Data analysis was performed using the computer software package SPSS (version 25, IBM Corp., Armonk, NY) and GraphPad Prism version 10.0.0 for Mac OS, GraphPad Software, Boston, Massachusetts USA, www.graphpad.com”. Unpaired categorical and binary variables were analyzed in contingency tables using Fisher’s exact test. For non-normally distributed variables, continuous variables were summarized as median and range, normally distributed variables as mean ± SD and categorical variables as absolute and percentage values. For the comparison of continuous variables, the Mann–Whitney U-test was chosen because the data were predominantly not normally distributed. OS was analyzed by the Kaplan–Meier method using Gehan–Breslow–Wilcoxon test. The hazard ratio was calculated using the Mantel-Haenszel test. Differences with an error probability of *p* < 0.05 were considered statistically significant. Finally, a stepwise backward method was used to construct a multivariate logistic regression model to analyze age, ECOG, KPS, MGMT, radio-, chemotherapy and EOR as predictors of PFS and OS.

The propensity score was generated using a logistic regression model. Age at diagnosis, number of lesions, location tumor volume, EOR and adjuvant treatment were used as covariates.

### Ethical approval

Data acquisition and analysis were performed anonymously and were approved by the Ethics Committees of the Medical Association of Rhineland Palatinate and Bavaria, Germany. According to local laws, further consent is not necessary for retrospective analysis.

## Results

### Baseline characteristics

A total of 483 patients with newly diagnosed glioblastoma (CNS WHO grade 4) were assessed for ML glioblastomas. 134 patients had multiple lesions (72 MF, 62 MC) and 349 had unifocal tumors. Mean age at diagnosis was 63.8 ± 0.7 years, and 47.7% were female. The median follow-up was 7.1 ± 11.5 months (range = 0–144 months). The median ECOG score at the time of admission was 1 (1; SD = 0.87) and remained unchanged at the time of discharge (1; SD = 1.2). Methylation of the MGMT promotor was detected in 49.1% of all patients. All tumors were IDH 1/2 wildtype (Table 1). No differences in patient characteristics were detected (Supp. Table 1).

**Table 1:** Baseline demographics and histology

	Solitary lesion	Multifocal	Multicentric	Total
Patients (n)	349	72	62	483
Age (SE, range)	64.4 (0.7; 18.0–88)	63.0 (1.3; 36–84)	60.9 (1.7; 18–84)	63.8 (0.7; 18–84)
Sex female (%)	140 (46.1)	33 (55)	30 (48)	203 (47.7)
ECOG (range)	1 (0–4)	1 (0–3)	1 (0–3)	1 (0–4)
MGMT methylation (n, %)				
No	146 (59.8)	19 (50.0)	21 (52.5)	186 (57.8)
Yes	98 (40.2)	19 (50.0)	19 (47.5)	136 (49.1)
Not available	105	22 (-)	22 (-)	149 (-)
IDH-mutation (n, %)				
Wildtype	248 (100)	47 (100)	58 (100)	353 (100)
Mutant	0	0 (0)	0 (0)	0 (0)
Not available	101 (-)	25 (-)	4 (-)	150 (-)

### Radiology data

**Fig. 1:**
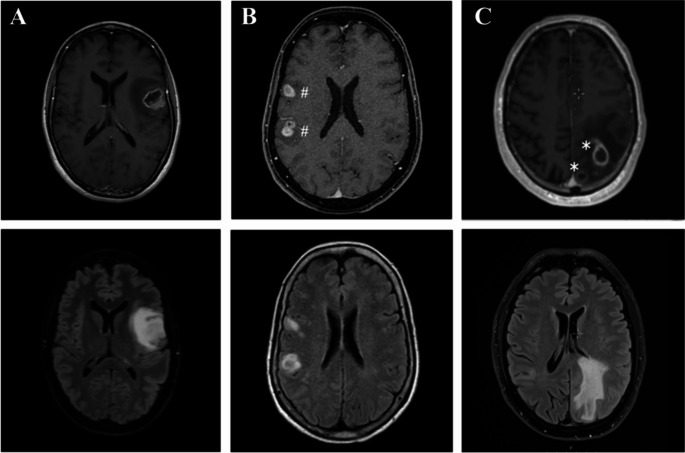
MRI T1 with Gadolinium contrast enhancement (upper) and T2 FLAIR (lower). Solitary lesion glioblastoma (**A**), multicentric glioblastoma without FLAIR interconnection (**B**) and multifocal glioblastoma (**C**)

Tumors with ML involved more than one lobe in 72.6% of all patients. Tumor locations were predominantly temporal (68.8%), followed by frontal (42.2%), parietal (38.5%), deep lesions (23.9%), insular (17.4%), occipital (14.7%), brainstem (4.6%) and posterior fossa (3.7%). Tumors with multiple lesions affected both hemispheres more frequently than tumors with SL (SL:6.6% MF: 35.0%, MC: 25.8%; *p* < 0.0001) The number of contrast-enhancing lesions was similar in MF (2.4 ± 0.66) and MC (2.2 ± 0.44) tumors (*p* = 0.59) (Fig. 1, Supp. Table 2).

**Table 2:** Univariate association of patient characteristics and treatment modality with progression-free and oerall survival

	Progression-free survival		Overall survival	
	HR (95% CI)	P value	HR (95% CI)	P value
MGMT methylation status	0.78 (0.512–1.17)	0.079	0.62 (0.39–0.97)	0.024
Extent of resection CR/Biopsy NTR/Biopsy STR/Biopsy PR/Biopsy CR/PR NTR/PR STR/PR	0.28 (0.10–0.81) 0.26 (0.08–0.80) 0.40 (0.09–0.80) 0.48 (0.19–1.19) 0.39 (0.18–0.84) 0.48 (0.23–0.99) 0.72 (0.38–1.37)	< 0.0003 < 0.0001 0.0068 0.05 0.0044 0.016 0.27	0.28 (0.14–0.56) 0.33 (0.17–0.63) 0.32 (0.17–0.62) 0.45 (0.24–0.83) 0.35 (0.19–0.65) 0.48 (0.27–0.88) 0.50 (0.28–0.88)	< 0.0001 < 0.0001 < 0.0001 < 0.01 < 0.0001 0.0078 0.0054
Radiotherapy (RT) hfRT/cRT	0.11 (0.004–3.17)	< 0.0001	0.13 (0.02–0.92)	< 0.0001
Adjuvant Therapy Stupp/Other	0.04 (0.28–0.86)	< 0.0001	0.44 (0.26–0.73)	< 0.0001

### Clinical data and demographics

Age (SL: 65.12 ± 12.32 years, MF: 62.88 ± 11.89 years, MC: 60.90 ± 13.43; *p* = 0.99), sex (SL: 42.1%, MF: 43.9% females, MC: 48.4%; *p* = 0.71) and ECOG at the time of admission (SL: 1 (IQR 1–2), MF: 1 (IQR: 1–2), MC: 1 (IQR: 1–2); *P* > 0.99) did not differ between patients with SL, MF and MC tumors. ECOG score at discharge was similar in patients with SL (Median: 1, Range: 0–5) compared to MF (Median: 1, Range: 0–5) lesions. MGMT promotor methylation was found in 49.5% of patients with SL, 43.9% in MF and 48.9% in those with MC tumors (*p* = 0.768).

### Survival in SL and ML glioblastoma

**Fig. 2:**
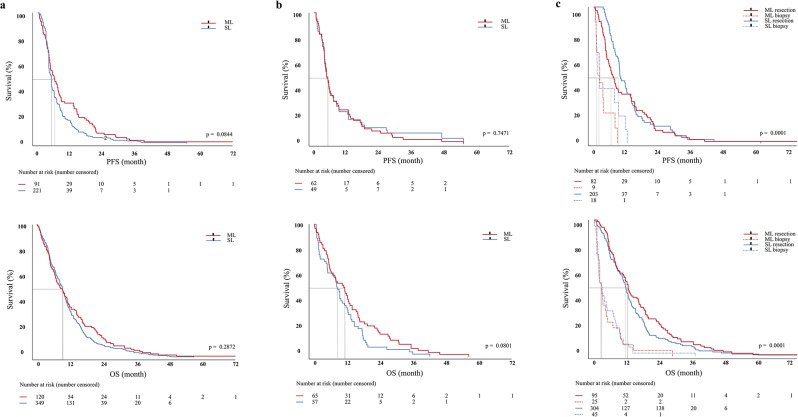
Survival of Glioblastoma with solitary and multiple lesions: **a**) The mean PFS (*p* = 0.084) and OS (*p* = 0.287) are similar in SL and ML glioblastoma. **b**) To adjust for biological and treatment differences ML and SL tumors were matched for age, ECOG, EOR, MGMT promotor methylation and adjuvant treatment according to the Stupp protocol using propensity score matching. The median PFS and OS of patients with ML (4.86 months, 95%CI 0.67–1.4 and 10.9 months, 95%CI 0.87–1.76 respectively) was statistically not different from those with SL (4.86 months, 95%CI 0.71-1.50- and 9.11-months 95%CI 0.57–1.15 respectively) (*p* = 0.08). **c**) Surgical resection was associated with improved survival in both groups compared to biopsy (*p* < 0.0001)

The mean PFS was 7.1 months (SE: 1.1 months, 95% CI: 6.19–08.09) in SL and 10.4 months (SE: 1.5 months, 95% CI: 7.4–13.3) in ML glioblastomas (*p* = 0.0844). Mean OS did not differ between SL (12.7 months, SE: 0.6 months, 95% CI: 11.5–13.9) and ML (14.5 months, SE: 1.5 months, 95% CI: 11.5–17.4) tumors (*p* = 0.2872) (Fig. 2a).

To adjust for biological and treatment differences ML and SL tumors were matched for age, ECOG, EOR, MGMT promotor methylation and adjuvant treatment according to the Stupp protocol using propensity score matching. Mean difference between both groups was reduced from 0.5 to 0.1 on average for all matching variables. The median PFS and OS of patients with ML (4.86 months, 95%CI 0.67–1.4 and 10.9 months, 95%CI 0.87–1.76 respectively) was statistically not different from those with SL (4.86 months, 95%CI 0.71-1.50- and 9.11-months 95%CI 0.57–1.15 respectively) (*p* = 0.08) (Fig. 2b).

Surgical resection was associated with improved survival in both groups compared to biopsy (SL: 16.7 ± 1.8 months and 4.9 ± 1.2 month; ML: 12.6 ± 0.8 months and 5.0 ± 0.9 months, *p* < 0.0001) (Fig. 2c).

### Survival in MC and MF glioblastoma

**Fig. 3:**
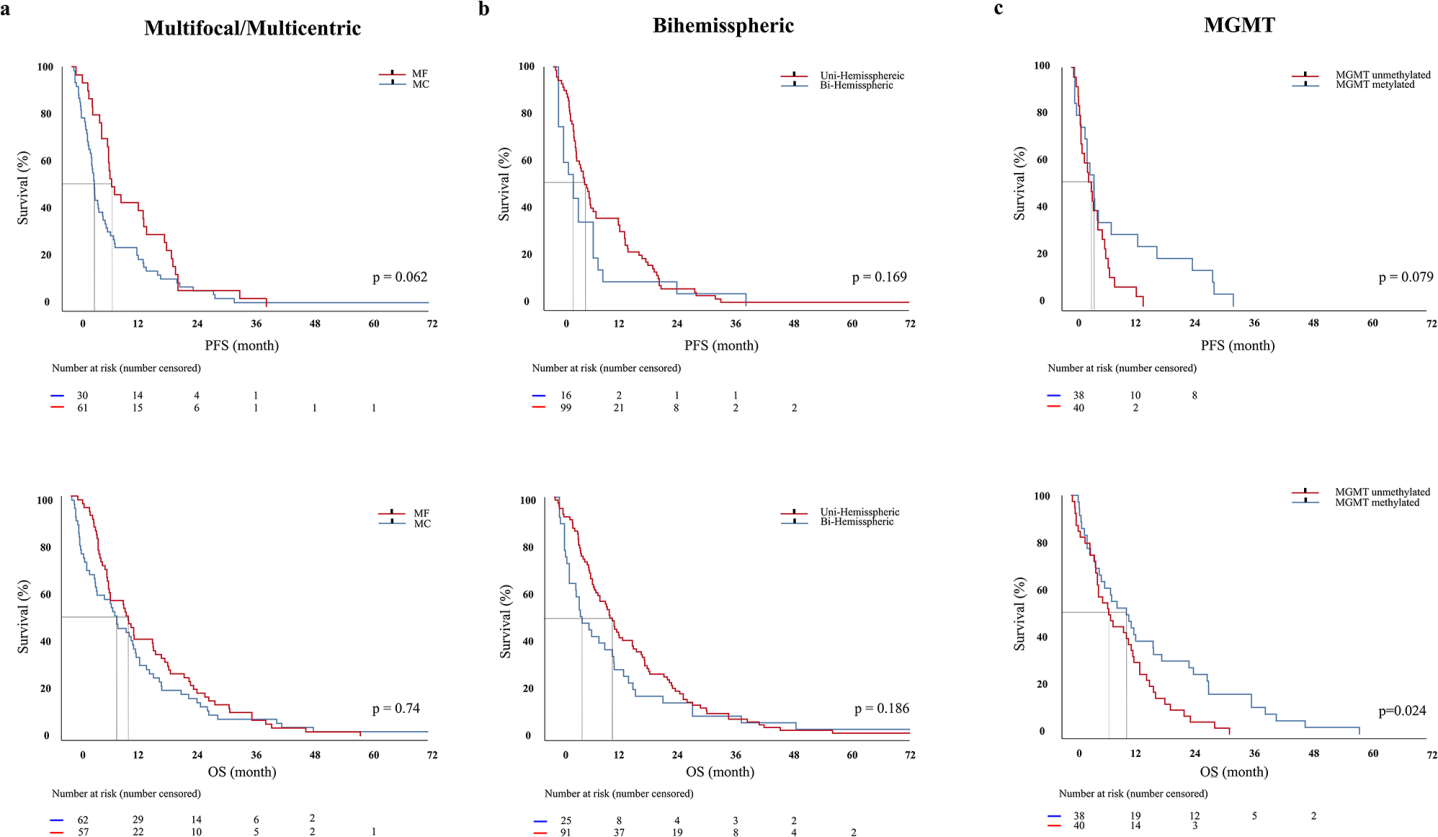
Univariate association of multiple lesions, hemisphere involvement and MGMT status with progression-free (upper) and overall survival (lower). MF and MC have similar PFS (*p* = 0.062) and OS (*p* = 0.74) (**a**). Involvement of both hemispheres is not associated with impaired outcome (*p* = 0.186), while MGMT methylation is a predictor for prolonged OS (*p* = 0.024) (**b + c**)

The mean PFS was 12.4 months (SE: 1.67 months, 95% CI: 7.2–15.1) in MF and 9.4 months (SE: 2.0 months, 95% CI: 3.9–6.3) in MC glioblastomas (*p* = 0.62). Mean OS did not differ between MF (15.4 months, SE: 1.6 months, 95% CI: 7.5–16.6) and MC (13.5 months, SE: 2.6 months, 95% CI: 4.8–12.6) tumors (*p* = 0.74). For all tumors with multiple lesions combined mean PFS was 10.4 months (SE: 1.5 months, 95% CI: 4.5–7.7), mean OS was 14.5 months (SE: 1.5 months, 95% CI: 7.5–13.6). Tumor occurrence in both hemispheres is not associated with an impaired PFS (HR: 0.72, 95% CI: 0.41–1.24, *p* = 0.169) and OS (HR: 0.78, 95% CI: 0.512–1.17, *p* = 0.186). Moreover, PFS is independent (HR: 0.62, 95% CI: 0.34–1.11, *p* = 0.079), while OS is dependent (HR: 0.62, 95% CI: 0.39–0.97, *p* = 0.024) on MGMT promotor methylation status. It is of note that PFS and OS in patients with multiple lesions were independent of age (r^2^ = 0.021) and KPS (r^2^ = 0.054) (Table 2; Fig. 3).

### Surgical data

The decision for surgical resection of ML cases was based on clinical status (KPS > 70) and tumor location in relation to eloquent areas. CR was achieved in 19 (15.6%) patients (MF: 11/18.3%; MC: 8 /13.9%), NTR in 20 (16.4%) patients (MF: 8/13.3%; MC: 12/19.4%), STR in 32 (26.2%) patients (MF: 11/18.3%; MC: 8/33.9%), PR in 26 (21.3%) patients (MF: 11/18.3%; MC: 8/12.9%)) and 25 (20.5%) underwent biopsy only (MF: 21/35%; MC: 4/6.5%). Functional outcome after surgery was similar in MF and MC glioma. It improved in 35 patients (32.1%), was unchanged in 49 (45.0%) and deteriorated in 25 (22.9%). The mean KPS before and after surgery remained 70 in both groups and no differences in procedure related complications were detected (Supplement Table 3).

**Table 3:** Multivariate analysis of patient characteristics and treatment modality association with progression-free and overall survival

	Progression-free survival		Overall survival	
	HR (95% CI)	P value	HR (95% CI)	P value
Age	0.99 (0.959–1.032)	0.7604	1.0 (0.97–1.04)	0.8575
Tumor Vol.	1.00 (0.987–1.021)	0.6515	0.99 (0.97–1.0)	0.0386
KPS	0.99 (0.9670–1.019)	0.5585	0.97 (0.95–0.99)	0.0066
Resection/Biopsy	8.44 (2.272–31.35)	0.0013	2.6 (0.67–8.7)	0.1521
EOR				
**CR**	0.43 (0.109–1.583)	0.2091	0.15 (0.03–0.59)	0.0089
**NTR**	1.05 (0.375–2.90)	0.9208	0.37 (0.14–0.93)	0.0358
**STR**	0.45 (0.137–1.491)	0.1911	0.26 (0.079–0.79)	0.0201
**Biopsy**	4.38 (0.66–24.80)	0.1046	1.15 (0.32–4.58)	0.8403
Chemo	1.01 (0.378–2.934)	0.9878	0.38 (0.1–1.47)	0.1603
Stupp	1.40 (0.090–20.56)	0.8049	3.50 (1.28–10.79)	0.0196
Radiation	0.96 (0.916–1.005)	0.0777	1.01 (0.088–9.38)	0.9935
Radiation dose	0.66 (0.306–1.410)	0.2833	0.97 (0.93–1.01)	0.1267
MGMT methylation	1.13 (0.469–2.904)	0.7976	1.70 (0.83–3.64)	0.1574
Bihemispheric	0.99 (0.959–1.032)	0.2091	0.42 (0.19–0.96)	0.0378

The EOR was a significant predictor of PFS and OS in patients with multiple lesions. The median PFS for CR was 15 months (SE: 2.8 months 95% CI 8.9–21.1), for NTR 13.5 months (SE: 2.5 months, 95% CI 8.2–18.7), for STR 11.5 months (SE: 4.0 months, 95% CI 3.2–19.8), for PR 6.7 months (SE: 1.5 months, 95% CI 3.5–10.0) and for biopsy 3.2 months (SE: 5.4 months, 95% CI 1.0–5.4).

The median OS for CR was 21.6 months (SE: 2.6 months 95% CI 16.3–27.0), for NTR 18.8 months (SE: 3.4 months, 95% CI 9.6–27.7), for STR 18.6 months (SE: 4.5 months, 95% CI 9.6–27.7), for PR 9.2 months (SE: 1.3 months, 95% CI 6.4–11.9) and for biopsy 4.7 months (SE: 1.4 months, 95% CI 2.2–1.2).

**Fig. 4:**
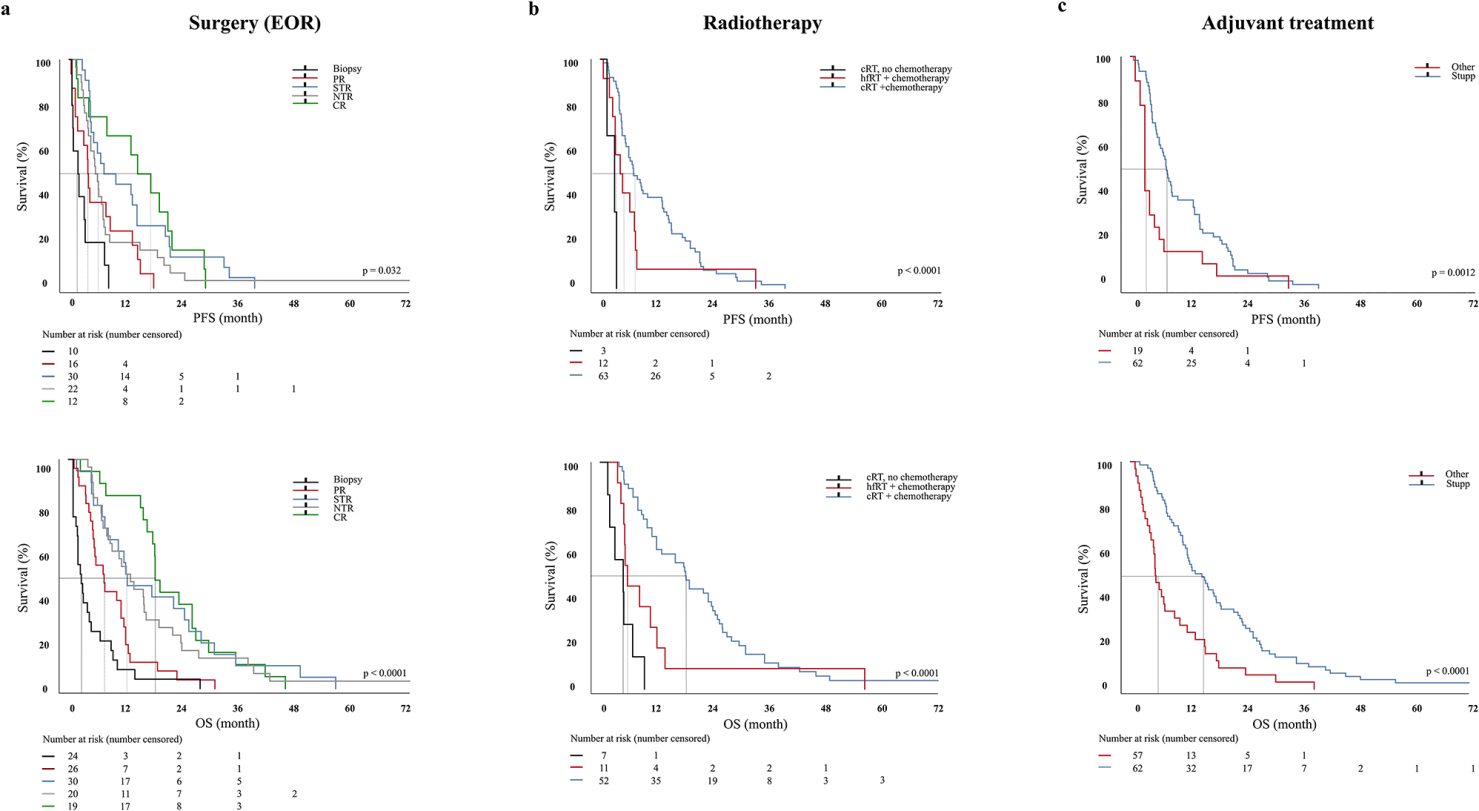
Univariate association of surgery (EOR), radiotherapy and adjuvant treatment with progression-free (upper) and overall survival (lower). CR, NTR, and STR significantly prolonged PFS (HR: 0.28, 95% CI: 0.10–0.81, *p* < 0.0003; HR 0.26, 95% CI: 0.08–0.80, *p* < 0.0001; and HR: 0.40, 95% CI: 0.09–0.80, *p* = 0.0068 respectively) and OS (HR: 0.28, 95% CI: 0.14–0.56, *p* < 0.0001; HR 0.33, 95% CI: 0.17–0.63, *p* < 0.0001 and HR: 0.32, 95% CI: 0.17–0.62, *p* = 0.0068) compared to biopsy alone (**a**). cRT was associated with prolonged PFS (HR: 0.11, 95% CI: 0.004–3.17, *p* < 0.0001) while both, cRT and hfRT results to prolonged OS (HR: 0.41, 95% CI: 0.13–1.29, *p* = 0.045; HR: 0.13, 95% CI: 0.02–0.93, *p* < 0.0001) (**b**). Treatment according to the Stupp protocol was associated with prolonged PFS (HR: 0.49, 95% CI: 0.28–0.86, *p* = 0.0012) and OS (HR: 0.44, 95% CI: 0.26–0.73, *p* < 0.0001) (**c**)

CR, NTR, and STR significantly prolonged PFS (HR: 0.28, 95% CI: 0.10–0.81, *p* < 0.0003; HR 0.26, 95% CI: 0.08–0.80, *p* < 0.0001; and HR: 0.40, 95% CI: 0.09–0.80, *p* = 0.0068 respectively) and OS (HR: 0.28, 95% CI: 0.14–0.56, *p* < 0.0001; HR 0.33, 95% CI: 0.17–0.63, *p* < 0.0001 and HR: 0.32, 95% CI: 0.17–0.62, *p* = 0.0068 respectively) compared to biopsy alone. To a lesser degree, also PR did increase PFS (HR: 0.48, 95% CI: 0.19–1.19, *p* < 0.05) and OS (HR: 0.45, 95% CI: 0.24–0.83, *p* < 0.01). CR, NTR, and STR were superior to PR to increase PFS (*p* = 0.0327) and OS (*p* < 0.0001) (Table 2; Fig. 4A).

### Radiotherapy and multimodal adjuvant treatment data

Treatment decisions for adjuvant therapy were based on clinical status and histopathological findings, including molecular markers taking current treatment guidelines into account. Radiation treatment was performed in all patients with a good to moderate ECOG status (0–3). A total of 95 (70.9%) patients received radiotherapy (MF: 49 (68.1%); MC: 46 (74.2%)). Hypofractionated radiation (hfRT, 30–40 Gy) in patients older than 70 years, conventional radiation (cRT, 60 Gy) in younger patients or patients with exceptional health status. Of those patients receiving radiotherapy, cRT was used in 75.7%, hfRT in 24.3%. cRT was associated with prolonged PFS (HR: 0.11, 95% CI: 0.004–3.17, *p* < 0.0001) while both, cRT and hfRT results to prolonged OS (HR: 0.41, 95% CI: 0.13–1.29, *p* = 0.045; HR: 0.13, 95% CI: 0.02–0.93, *p* < 0.0001) (Fig. 4B).

Concomitant treatment using temozolomide (TMZ, 75mg/m^2^) during conventional radiotherapy (cRT), followed by 6 cycles of adjuvant TMZ (150-200mg/m^2^) for 5 days out of 28 days (Stupp protocol) was applied in about half of all patients (51.9%) independent of the MGMT promotor methylation status. Other adjuvant treatments included different agents such as lomustine (CCNU), bevacizumab or procarbazine (14.0%), as well as other regimens (one week on/one week off) or sequential therapy (2.3%). Chemo- (7.8%) or radiotherapy (6.2%) alone was used in a minority of cases. Adjuvant therapy was omitted in favor of a best supportive care (BSC) in patients who initially underwent biopsy alone (17.8%). The decision to biopsy followed by BSC was stratified by age, pre-operative KPS and respectability of the initial tumor. Treatment according to the Stupp protocol was associated with prolonged PFS (HR: 0.49, 95% CI: 0.28–0.86, *p* = 0.0012) and OS (HR: 0.44, 95% CI: 0.26–0.73, *p* < 0.0001) (Fig. 4C). Eventually, tumor recurrence was observed in all patients.

### Multivariate analysis

Variables associated with statistically significant effects on PFS or OS (MGMT promotor methylation, EOR, Stupp therapy, radiation therapy, bi-hemispheric involvement) and established variables (age, KPS, tumor volume) were included in a multivariate survival analysis. Here, performance status (HR: 0.97, 95% CI: 0.95–0.99, *p* = 0.006), EOR (CR: HR 0.15, 95%CI: 0.03–0.59, *p* = 0.0089; NTR: HR 0.37, 95%CI: 0.14–0.93, *p* = 0.0358; CR: STR 0.26 95%CI: 0.079–0.79, *p* = 0.0201) and concomitant treatment (HR: 3.5, 95% CI: 1.28–10.79, *p* = 0.019) remained statistically significant factors for survival (Table 3).

## Discussion

Glioblastomas with ML are considered different from those with SL in regard to their biological nature, clinical course and treatability [6, 8]. As the incidence of ML is noticeably lower than that of SL data on this distinct subtype remains scarce [23, 24]. Subdivision in MC and MF reflects the primary diffuse disseminated nature of ML tumors. ML are either present at initial diagnosis or develop later in the disease with synchronous occurrence of MF and MC lesions reported [25, 26]. Further, different histological entities, molecular landscapes and gene expression phenotypes can be found in parallel in ML gliomas [27]. It is thought that these differences in nature and dissemination result in a worse prognosis than those with SL tumors [28, 29]. As of today, there are no common treatment guidelines for ML tumors [29]. Despite little evidence, the notorious assumption of a worse prognosis often leads to limited treatment with focus on palliative care in early stages of the disease. These restrictions might no longer be feasible with growing insight into the pathomechanisms, clinical course and importance of multimodal therapy in ML glioblastoma [9, 16]. It is the aim of this study to gather insight into the clinical course of MC and MF glioblastoma and to substantiate the impact of multimodal therapy in tumors with ML in the largest cohort reported in literature.

Despite the efforts spent on characterization and radiologic differentiation of MF and MC tumors compared to SL glioblastoma, little is known on their respective clinical course. The mean age of 64 years of the present study is consistent with reports of other MF and MC glioblastoma [30]. Age distribution of classical SL glioblastoma and MF and MC glioblastoma have been reported to be similar [30]. While patients with formerly IDH1/2 wildtype tumors WHO grade II and III are younger (45 years), those with WHO grade IV tumors tend to be older (IDH1/2 wt astrocytoma with molecular features of a WHO grade IV tumor: 58 years; IDH1/2 wt glioblastomas: 55 years) [31, 32]. In concordance with molecular observations, MF and MC glioblastoma are similar to IDH1/2 wt glioblastoma with SL and fit into the expected age of diagnosis. MF and MC tumors occurred equally distributed in females and males (51/49%). Sex distribution differs as malignant gliomas occur more frequently in males, while diffuse gliomas are non-sex-specific [31, 32]. Median ECOG performance status was good and remained unchanged before (ECOG 1) and after surgery (ECOG 1) in our data set. Similar good performance indices before and after are reported in other cohorts with MF and MC glioblastomas [29, 32].

A direct comparison of MF and MC tumors in literature is missing. Both entities are usually subsumed as one albeit missing a defining clinical characterization and comparison [9, 16]. There is evidence that OS is dependent on lesion localization and distribution rather than MF or MC occurence [23, 24, 28]. However, in the presented cohort the clinical course is undistinguishable between both entities. The median number of lesions is 2 in both subgroups. It is of note that involvement of both hemispheres and the total number of lesions had no impact on clinical prognosis. This falls in line with the more recent recognition of the importance of tumor burden and occurrence of deep seated lesions rather than the exact number or general hemispheric distribution [23]. MC lesions localization and occurrence is related to migratory processes in an attempt to escape hypoxia and to reach oxygen-rich areas adjacent to blood vessels [29, 33]. Further, MC lesions might differ genetically representing a rather metachronous, independent glioma development [13]. On a molecular level MF tumors harbor higher frequencies of EGFR mutation and co-occurrence of EGFR/PTEN alterations, TERT and p53 [4, 12]. The high incidence of genetic alterations in key pathways are thought to contribute to a highly malignant phenotype [4, 12, 13]. It is of note that in the present analysis the highly malignant molecular phenotype did not reflect in the clinical course of either MF or MC glioblastoma. Identification of EGFR mutations and the EGFR-variant III (EGFRvIII) is becoming increasingly common as molecular targets for salvage therapy [34]. In IDH-wt glioblastoma response to alkylating chemotherapy is significantly better when the MGMT promoter is methylated [35]. Promoter methylation is detected in about 40% of all IDH-wt tumors [36, 37]. This proportion is matched by 48.7% promotor methylation present in this cohort. Here, the MGMT promotor methylation status is not different between MF and MC tumors. In analogy to SL tumors, MGMT promotor methylation is a significant prognostic factor of therapeutic response in ML glioblastoma. This observation is confirmed by data reported previously [24]. Taken together, there is mounting evidence that, despite being different in their respective genetic background, glioblastoma with MF and MC lesions behave clinical similar and can be treated as ML tumors rather than separate entities.

In previous publications, OS of tumors with ML ranges from 8.3 to 11.5 months [38]. Only 17% of all patients are thought to survive one year or longer [6, 7]. The importance of the EOR of SL glioblastomas has been demonstrated in many studies and many efforts have been made and intraoperative techniques developed to achieve a safe and CR or even supramarginal resection [39, 40]. Using matched pair analysis, PFS and OS in glioblastoma with SL and ML are indifferent in the present analysis. This stands in stark contrast to the perceived impaired prognosis of these patients [6, 7]. One reason might be the perception triggered undertreatment offering these patients only limited surgical interventions and early best supportive care [9, 16]. However, the presented data offers evidence that maximal safe resection and multimodal therapy are similarly effective in tumors with multiple lesions and substantially impacts overall prognosis. Multimodal therapy in patients with ML results in similar outcome and OS compared to matched patients with SL. Together, these findings argue for a more aggressive treatment affirming approach in the management of ML glioblastoma.

As unified guidelines for multimodal treatment of ML glioblastomas are missing, uncertainty remains regarding surgical resection and best medical treatment. The role of surgical resection in these tumors is still a matter of debate. So far, no conclusive data exist regarding the effect of the EOR on survival in ML glioblastoma per se and MF and MC tumors in particular. Previously, a smaller study of 34 patients receiving either biopsy or resection provided first evidence for a benefit from resection for greater median OS but not PFS despite the aggressive nature of these tumors [16]. In addition, a later study in 100 patients observed a significant difference between PFS and OS after GTR, STR and PR compared to biopsy [9]. Both studies subsumed MC and MF tumors without differential analysis [9, 16]. Although MC and MF tumors are thought to be of different genetic origin, similar therapeutic response can only be inferred. The presented results fall in line with the previously published data in terms of increased survival with larger EOR in MC and MF. Here, no statistically significant difference with resection rates between MF and MC tumors was detected. In all, despite the added complexity associated with the molecular era, the best available evidence supports maximal, but safe, resection for malignant glioma [41]. These findings substantiate the significance of greater EOR for longer OS in ML glioblastoma and extends the truthfulness of this paradigm to MC and MF glioblastoma. It is of note that in the presented cohort any degree of greater EOR improved survival without adding clinical deficit or impairment. However, it has not yet been possible to answer how much tumor volume needs to be removed in ML glioblastoma to make a decisive difference in PFS and OS. Here, we show that already subtotal resection with a cut-off value of 89% can make a crucial difference for survival of more than 12 months.

Glioblastoma is a highly invasive tumor rendering radical tumor resection not curative. It is thought that glioblastoma contain different populations of glioblastoma stem cells mediating tumor recurrence [42]. Adjuvant treatment is therefore mandatory whenever possible. Depending on patients age, the current standard of care consists of concomitant daily temozolomide, and radiotherapy followed by temozolomide cycles [2]. The standard of care treatment regimen (Stupp protocol) consists of radiation treatment (60 Gy) with concurrent temozolomide (TMZ) (75 mg/m^2^) followed by 6 cycles of adjuvant TMZ (150–200 mg/m2 (Stupp protocol) [2]. In those patients of younger age and methylated MGMT promotor, CCNU can be added during first-line therapy [43]. For those over the age of 70 years, concomitant-, sequential- or monotherapy using either RT or alkylating chemotherapy, are considered [44, 45]. Apart from the EOR, the decision for adjuvant treatment has not been addressed in any larger studies so far. Evidence in literature for the benefit of radiation and chemotherapy in ML glioblastoma is scarce. Treatment decisions can only be inferred from SL strategies. The proportion of patients who received concomitant radiochemotherapy after surgery was 75.7%, of those with RT alone was 14.3% and chemotherapy alone was 10%. This has been similarly reported in the few studies that have been published on this specific tumor cohort [5]. Treatment according to the Stupp protocol was associated with significantly prolonged PFS and OS in MF and MC tumors [5]. In a smaller study of 11 patients, whole brain radiation instead of standard radio therapy was found to be well tolerated with stipulated benefit on survial [19]. Additionally, both standard RT and hf RT led to a longer OS in ML glioblastoma [7]. Hypofractionated RT is supposed to reduce neurotoxicity while maintaining anti-tumoral activity [46, 47]. The results presented here demonstrate the biological activity of standard RT and hf RT in the context of ML glioblastoma.

## Conclusion

For the first time, the presented data provides conclusive insight into the influence of multimodal treatment in patients with MC or MF glioblastoma with predominantly not more than two lesions. By fully using the current means of treatment in MF und MC tumors, equal survival rates compared to patients with SL tumors can be achieved. No difference was observed among the two subtypes of MF and MC tumors in regard to PFS and OS, when equally treated. The results reflect the importance of an equally aggressive maximal treatment effort in this particular and often marginalized group of patients.

## Limitations

The study has several limitations. It is limited by its retrospective, non-randomized design. As inherent to retrospective surgical analysis, the role of selection bias between biopsy and resection is a potential source of confounding variables. It is likely that favorable tumor location, such as non-eloquent location, may have conferred improved survivability. Additionally, older patients or those with clinical signs of frailty or impairment might have been selected for biopsy due to concerns over the ability to tolerate a long surgery with extensive resection.

## Electronic Supplementary Material

Below is the link to the electronic supplementary material.

Supplementary Material 1

## Data Availability

No datasets were generated or analysed during the current study.
