# Supplementary material for: Multimodal treatment of glioblastoma with multiple lesions - a multi-center retrospective analysis
Source: J Neurooncol. 2024 Nov 19;170(3):555–66. doi: 10.1007/s11060-024-04810-3 (PMC11614972; doi:10.1007/s11060-024-04810-3)
Supplement: Supplementary file 1 — Supplementary Material 1 [file 11060_2024_4810_MOESM1_ESM.docx]

Supp. Table 1 Preoperative Clinical Characteristics

|  | Multifocal | Multicentric | Total |
| --- | --- | --- | --- |
| General symptoms |  |  |  |
| Seizures | 9 (19.2 %) | 16 (25.8 %) | 15 (13.8 %) |
| Headache | 14 (29.8 %) | 16 (25.8 %) | 30 (27.5 %) |
| Confusion | 15 (31.9 %) | 8 (12.9 %) | 23 (21.1 %) |
| Nausea/vomit | 4 (8.5 %) | 7 (11.3 %) | 11 (10.1 %) |
| Urinary incontinence | 1 (2.1 %) | 1 (1.6 %) | 2 (1.8 %) |
| Cognitive decline | 15 (31.9 %) | 13 (20.1 %) | 28 (25.7 %) |
| Focal neurological deficits |  |  |  |
| Motor | 14 (29.8 %) | 16 (25.8 %) | 30 (27.5 %) |
| Sensory | 3 (6.4 %) | 5 (8.1 %) | 8 (7.3 %) |
| Speech | 12 (25.5 %) | 28 (45.1 %) | 40 (36.7 %) |
| Visual | 5 (10.6 %) | 13 (21.0 %) | 18 (16.5 %) |
| Cranial nerve palsy | 7 (14.9 %) | 5 (8.1 %) | 12 (11.0 %) |
| Higher functions | 6 (12.8 %) | 10 (16.1 %) | 16 (14.7 %) |
| Gait disturbance and vertigo | 7 (14.9 %) | 10 (16.1 %) | 17 (15.6 %) |
| Median preoperative KPS | 70 | 70 | 70 |

Supp. Table 2. Radiological and Histological Findings

|  | Multifocal | Multicentric | Total |
| --- | --- | --- | --- |
| Hemisphere |  |  |  |
| Single hemisphere | 38 (80.9 %) | 46 (74.2 %) | 84 (77.1 %) |
| Midline | 21 (44.7 %) | 28 (45.2 %) | 49 (45.0 %) |
| Both hemispheres | 9 (19.1) | 16 (25.8 %) | 25 (22.9 %) |
| Posterior fossa | 5 (10.6 %) | 4 (6,5 %) | 9 (8.3 %) |
| No of lesions |  |  |  |
| 2 lesions | 38 (80.9 %) | 51 (82.3 %) | 89 (81.7 %) |
| 3 lesions | 6 (12.8 %) | 10 (16.1 %) | 16 (14.7 %) |
| 4 lesions | 2 (4.3 %) | 1 (1.6 %) | 3 (2.8 %) |
| 5 lesions | 0 (0 %) | 0 (0 %) | 0 (0 %) |
| Lobes involved |  |  |  |
| Frontal | 19 (40.4 %) | 27 (43.6 %) | 46 (42.2 %) |
| Parietal | 16 (34.0 %) | 26 (41.9 %) | 42 (38.5 %) |
| Temporal | 39 (83.0 %) | 36 (58.0 %) | 75 (68.8 %) |
| Occipital | 6 (12.8 %) | 10 (16.1 %) | 16 (14.7 %) |
| Insular | 10 (21.3 %) | 9 (14.5 %) | 19 (17.4 %) |
| Cerebellum | 1 (2.1 %) | 3 (4.8 %) | 4 (3.7 %) |
| Brainstem | 4 (8.5 %) | 1 (1.6 %) | 5 (4.6 %) |
| Deep lesions | 18 (38.3 %) | 8 (12.9 %) | 26 (23.9 %) |
| No. of eloquent areas involved |  |  |  |
| 0 | 3 (6.4 %) | 32 (51.6 %) | 35 (32.1 %) |
| 1 | 23 (49.0 %) | 16 (25.8 %) | 39 (35.8 %) |
| 2 | 16 (34.0 %) | 12 (19.3 %) | 28 (25.7 %) |
| 3 | 5 (10.6 %) | 1 (1.6 %) | 6 (5.5 %) |
| Corpus callosum invasion | 22 (46.8 %) | 15 (25.2 %) | 37 (33.9 %) |
| Midline shift (>5mm) | 25 (53.2 %) | 28 (45.2 %) | 53 (48.6 %) |
| Mean tumor volume (cm^3^) (range) | 50.4 (5.0-151) | 36.6 (5.4-157.3) | 43.5 (5.0-157.3) |
| MGMT hypermethylation |  |  |  |
| No | 10 (21.3 %) | 21 (33.9 %) | 31 (28.4 %) |
| Yes | 6 (12.8 %) | 19 (30.6 %) | 25 (22.9 %) |
| Not available | 31 (66.0 %) | 22 (35.5 %) | 33 (30.3 %) |
| IDH-mutation |  |  |  |
| wildtype | 47 (100.0 %) | 56 (90.3 %) | 103 (94.5 %) |
| mutant | 0 (0 %) | 2 (3.2 %) | 2 (1.8 %) |
| Not available | 0 (0 %) | 4 (6.5 %) | 4 (3.7 %) |

|  | Multifocal | Multicentric | Total |
| --- | --- | --- | --- |
| Postoperative evaluation |  |  |  |
| Neurological evaluation |  |  |  |
| Unchanged | 25 (53.2 %) | 24 (38.7 %) | 49 (45.0 %) |
| Improved | 11 (23.4 %) | 24 (38.7 %) | 35 (32.1 %) |
| Worsened | 11 (23.4 %) | 14 (22.6 %) | 25 (22.9 %) |
| Postoperative KPS |  |  |  |
| Unchanged | 31 (66.0 %) | 26 (42.0 %) | 57 (52.2 %) |
| Improved | 8 (17.0 %) | 18 (29.0 %) | 26 (23.9 %) |
| Worsened | 8 (17.0 %) | 18 (29.0 %) | 26 (23.9 %) |
| Mean KPS (range) | 70 (60-80) | 70 (60-80) | 70 (60-80) |
| Perioperative complication |  |  |  |
| Hemorrhage | 3 (6.4 %) | 3 (4.8 %) | 6 (5.5 %) |
| Deep vein thrombosis | 2 (4.3 %) | 1 (1.6 %) | 3 (2.8 %) |
| Pneumonia | 1 (2.1 %) | 0 (0 %) | 1 (0.9 %) |
| Seizures | 2 (4.3 %) | 3 (4.8 %) | 5 (4.6 %) |
| Systemic infection | 1 (2.1 %) | 1 (1.6 %) | 2 (1.8 %) |

Supp. Table 3. Postoperative Clinical Status and Complications
